# Establishment of a nomogram model based on immune-related genes using machine learning for aortic dissection diagnosis and immunomodulation assessment

**DOI:** 10.7150/ijms.100572

**Published:** 2025-01-21

**Authors:** Yanjun Hou, Yangyang Zhao, Zhensu Shi, Yipeng Pan, Kaijia Shi, Chaoyang Zhao, Shengnan Liu, Yongkun Chen, Lini Zhao, Jizhen Wu, Guangquan Ge, Wei Jie

**Affiliations:** 1 Department of Cardiovascular Surgery, the Second Affiliated Hospital, Hainan Medical University, Haikou 570311, China.; 2 Key Laboratory of Tropical Translational Medicine of Ministry of Education & Hainan Provincial Key Laboratory for Tropical Cardiovascular Diseases Research, School of Public Health, Hainan Medical University, Haikou 571199, China.; 3 Emergency and Trauma College, Hainan Medical University, Haikou 571199, China.; 4 Department of Transplantation, the Second Affiliated Hospital, Hainan Medical University, Haikou 570311, China.; 5 Department of Pharmacy, the Second Affiliated Hospital, Hainan Medical University, Haikou, 570311, China.; 6 Department of Quality Control, the Second Affiliated Hospital, Hainan Medical University, Haikou 570311, China.; Yanjun Hou and Yangyang Zhao contributed equally to this work.

**Keywords:** aortic dissection, immune-related genes, machine learning, nomogram models, diagnosis

## Abstract

The clinical manifestation of aortic dissection (AD) is complex and varied, making early diagnosis crucial for patient survival. This study aimed to identify immune-related markers to establish a nomogram model for AD diagnosis. Three datasets from GEO—GSE52093, GSE147026 and GSE153434—were combined and used for identification of immune-related causative genes using weighted gene co-expression network analysis, and 136 immune-related genes were obtained. Then, 15 pivotal genes were screened by the protein-protein interaction network. Through machine learning including the Least Absolute Shrinkage and Selection Operator algorithm, random forest algorithm, and multivariate logistic regression, four key feature genes were obtained—*CXCL1*, *ITGA5*, *PTX3*, and *TIMP1*—and the diagnostic scores based on these four genes were proved to be effective in distinguishing between AD patients and healthy donors. External dataset (GSE98770 and GSE190635) validation revealed this nomogram displayed strong predictive significance. Further analysis revealed that these genes are related with neutrophils, resting NK cells, resting mast cells, activated mast cells, activated dendritic cells, central memory CD4 T cells, γδ T cells, natural killer T cells, and myeloid-derived suppressor cells in AD. Finally, these four genes were validated to be upregulated in AD patients' tissue and serum samples compared with controls. These results suggest that this nomogram model, using machine learning identified four immune-related genes *CXCL1*, *ITGA5*, *PTX3*, and *TIMP1*, displays superior diagnostic ability in distinguishing AD and healthy individuals, and immune cells commonly associated with these hub genes may be therapeutic targets for AD.

## Introduction

Cardiovascular disease is the leading cause of death and disability worldwide in most developed and developing countries 1. Aortic dissection (AD) is a fatal disease with a high mortality rate that affects the complex three-layered wall of the aorta. The Stanford system, the most commonly used anatomical typing system, classifies AD into two types: type A thoracic AD (TAAD) involves the ascending aorta; and type B thoracic AD (TBAD) does not include the ascending aorta and spreads distally from the isthmus 2. In untreated acute TAAD, the initial mortality rate is about 1% per hour, a significant proportion of patients cannot survive beyond three days, and nearly 80% died within two weeks 3. The 30-day mortality rate for TBAD is 13.9% lower than for TAAD 4. Therefore, early diagnosis is crucial for intervention in AD.

The clinical presentation of AD is characterized by its complexity and variability. Patients often lack specific signs and symptoms. Clinicians look for common symptoms, such as chest and back pain; however, atypical symptoms in pain-free patients can present a diagnostic challenge, leading to potential misdiagnosis 5. Computed tomography (CT) angiography is commonly used to diagnose AD, but it is the last option for diagnosis, so clinicians have little time to plan surgery. Therefore, rapid identification of biomarkers is essential for the early diagnosis of AD. Recently, computational methods, particularly machine learning, have gained attention for their ability to predict events and support clinical decision-making 6. Machine learning is a branch of computer science that uses data patterns to identify and predict outcomes. It excels in situations involving many variables, especially when these relationships are complex and non-linear 7-9.

Several models for diagnosing AD have emerged in recent years. These include clustering-based integrated learning models for AD screening 10, chromatin regulatory factor-based diagnostic models 11, and multimachine diagnostic models 12. However, some models did not consider changes in the immune microenvironment associated with AD. Notably, aortic inflammation is a prominent feature of AD. Immune cell infiltration of the intima and adventitia leads to increased oxidative stress, inflammatory factors, and elevated expression of matrix metalloproteinases (MMPs). These factors lead to vascular smooth muscle cell (VSMC) apoptosis and aortic remodeling and play a vital role in the pathogenesis of AD 13. Immune infiltration occurs in the central and lateral perimeters of AD specimens and involves various cell types, such as neutrophils, mast cells, macrophages, and lymphocytes. Aortic immune cells show considerable heterogeneity, displaying different cellular states and functions in each population. In addition, the recruitment and activation of macrophages within the midcapsules are key events in the early stages of acute AD 14. Therefore, it is important to characterize AD based on the immune cells or immune function level.

To date, the mechanistic role of immune infiltration in AD remains unclear. In this study, Gene Expression Omnibus (GEO) datasets were used to explore the possible pathogenesis and analyze differential immune-related genes between healthy individuals and AD samples, then multiple machine learning algorithms were adopted to identify the hub genes, and a nomogram model was established. This study identified as an excellent performance in the diagnosis of AD. And the diagnostic model was verified by patient tissue and serum samples. These results provide novel machine-learning-based approaches for AD diagnosis and immunomodulation assessment.

## Materials and Methods

### Public data collection and de-batching

AD-related datasets were obtained from the GEO database (https://www.ncbi.nlm.nih.gov/geo/). The inclusion criteria were defined as follows: (1) the dataset samples were from aortic tissue; (2) the dataset included AD patients and unaffected controls; and (3) the dataset included human gene expression profiles. After a thorough review, we selected the GSE52093, GSE147026, GSE153434, GSE98770 and GSE190635 datasets. Each dataset was carefully examined to confirm it met our predefined criteria. The GSE52093 dataset comprised 7 AD patient samples and 5 control samples, the GSE147026 dataset included 4 patient samples and 4 control samples, and the GSE153434 dataset had 10 control samples and 10 patient samples. All data were converted to log_2_ format for subsequent analyses. This transformation involves applying a logarithmic function to the raw expression values. Next, we applied the Surrogate Variable Analysis (SVA) algorithm 15 to remove the batch effect in three of the gene expression profiling datasets, GSE52093, GSE147026, and GSE153434, and then merged these datasets into a single training dataset, which included 21 AD samples and 19 control samples. We apply SVA to three datasets to ensure that the results were comparable and not confounded by batch effects. GSE98770 and GSE190635 datasets were used for validation. Figure S1 depicts the relationship between the samples before and after removal of the batch effect.

### Identification of immune-related disease-causing genes

The R package “limma” with a significance threshold of *P* < 0.05 was used to identify differentially expressed genes (DEGs) between AD and control samples in the combined dataset 16. Differential gene expression data were visualized by volcano plots, graphs, and heat maps. We then assessed the availability of 1131 variant genes and constructed gene co-expression networks using the R package “WGCNA” 17. We chose a soft threshold of β = 10 to build these networks, targeting a scale-free R^2^ of 0.98. Subsequently, the neighbor-joining matrix was converted into a topological overlap matrix (TOM), which quantitatively describes the similarity of the nodes by comparing the weighted correlations between nodes and other nodes. Genes within the same module showed a high degree of co-expression, and the correlation between each module and the clinical data was calculated to identify clinically relevant modules.

### Gene enrichment analysis

Gene Ontology (GO) enrichment and Kyoto Encyclopedia of Genes and Genomes (KEGG) pathways were analyzed in R using the “clusterProfiler” software package to gain insight into the biological functions of the immune-related pathogenic genes 18.

### Protein-protein interaction networks

The protein-protein interaction (PPI) network was analyzed using STRING (https://string-db.org/) 19. To enhance the visual display of images downloaded from STRING and to identify important interacting genes, Cytoscape software and the MCODE plug-in were used 20. All genes capable of interacting in the PPI network were selected for further analysis. The top 30 important genes in the PPI network were predicted and explored using five topological analysis algorithms—DMNC, MCC, MNC, Degree, and EPC—provided by the cytoHubba plug-in for Cytoscape.

### Machine learning

Three algorithms were used to identify feature genes among the 15 pivotal genes. Least Absolute Shrinkage and Selection Operator (LASSO) is a regression method used for variable selection to increase prediction accuracy and improve the comprehensibility of statistical models 21. LASSO specific parameters: family = "binomial", nfolds = 10, and ten-fold cross-validation is used to adjust the optimal value of the parameter λ. The minimum lambda is defined as the optimal value. Support Vector Machine (SVM) is a powerful method to establish class boundaries and thus make label predictions based on single or multiple feature vectors 22. SVM specific parameters are: halfve. above = 20 and k = 10. and 10-fold cross validation is used to improve the accuracy of the algorithm performs well in high dimensional spaces and is able to handle nonlinear feature interactions. Random Forest (RF) is a suitable method for predicting continuous variables with minimal fluctuations because of its lack of restrictions on variable conditions, and superior accuracy, sensitivity, and specificity in predicting continuous variables and providing predictions 23. The RF parameters are as follows: ntree = 500, mtry = 3, importance = T, and the Gini index is used as an important measure. The Random Forest prediction is highly reproducible and the Random Forest algorithm was used to rank the DEGs based on the reduction of the Gini index and the top 15 genes with significant values greater than 3 were selected for downstream analysis. The diagnostic efficacy and optimal thresholds of these models were assessed by analyzing receiver operating characteristic (ROC) curves. This analysis included calculating the area under the ROC curve (AUC), sensitivity, specificity, positive predictive value (PPV), negative predictive value (NPV), accuracy, and their corresponding 95% confidence intervals (CIs).

### Constructing schematics and evaluating receiver operating characteristics

Nomogram of candidate genes was constructed using the R software package “rms” 24, where “score” represents the score of the candidate genes and “total score” is the cumulative score of all mentioned genes. A calibration curve was used to assess the prediction accuracy of the nomogram.

### External validation

GSE98770 and GSE190635 datasets were used for validation. The GSE98770 dataset contained 6 patient samples and 5 control samples, and the GSE190635 datasets contained 4 patient samples and 4 control samples. In the dataset, gene expression profiling was performed with mRNA and miRNA microarrays. In the validation dataset, the results of the predictive model are compared with the results of the training set to validate the model.

### Analysis of infiltrating immune cells

The immune microenvironment (IME) includes a variety of innate and adaptive immune cells, alongside stromal cells such as fibroblasts, lymphocytes, endothelial cells, adipocytes, inflammatory cells of bone marrow origin, blood vessels, a variety of signaling molecules, and extracellular matrix (ECM). Analyzing immune cell infiltration is critical to understanding disease progression and treatment response. The proportion of immune cells was quantified using the CIBERSORTx and ssGSEA algorithms 25. To further our understanding, we performed single sample gene set enrichment analysis (ssGSEA), an extension of the GSEA approach, creating 23 immune genomes. The immunological characteristics of all samples were assessed by ssGSEA using the “GSVA” R package 26. To analyze the level of the IME, we used the “ESTIMATE” software package 27.

### Ethics statement and clinical sample collection

A total of ten healthy serum and aortic samples were obtained from organ donors, and samples from 12 patients with AD were obtained from patients who underwent surgery at the Second Affiliated Hospital of Hainan Medical University from 2021 to 2022. The collected samples were cleaned, aliquoted, labeled, preserved in liquid nitrogen within 20 minutes of vascular resection, and processed rapidly. CT angiography was performed to assess the status of AD. The use of human blood and tissue was approved by the Institutional Review Board of the Second Affiliated Hospital of Hainan Medical University (approval number, 2021-011-03), and all methods were performed in accordance with the relevant guidelines.

### RNA extraction and qRT-PCR

Total RNA extraction was performed using a RNeasy Micro Kit (Qiagen, 74104). Subsequently, cDNA synthesis was performed using the QuantiTect Reverse Transcription Kit (Qiagen, 205311). Quantitative real-time polymerase chain reaction (qRT-PCR) was performed using SYBR Green qPCR Master Mix (Bio-Rad,1725110). The primer (5′-3′) sequences for qPCR were as follows: *CXCL1* forward, AGCTTGCCTCAATCCTGCATCC; *CXCL1* reverse, TCCTTCAGGAACAGCCACCAGT; *ITGA5* forward, GCCGATTCACATCGCTCTCAAC; *ITGA5* reverse, GTCTTCTCCACAGTCCAGCAAG; *PTX3* forward, CGAAATAGACAATGGACTCCATCC; *PTX3* reverse, CTCATCTGCGAGTTCTCCAGCA; *TIMP1* forward, GGAGTGTCTGCGGATACTTC; *TIMP1* reverse, GCAGGTAGTGATGTGCAAGAGTC; *β-actin* forward, CACCATTGGCAATGAGCGTTC; and *β-actin* reverse, AGGTCTTTGCGGATGTCCACGT.

### Protein extraction and western blotting

Aortic tissue was collected in 1.5 ml Eppendorf tubes. Proteins were extracted by adding 300 ml of 2% SDS loading buffer to 1 mg of ground tissue supplemented with β-mercaptoethanol. Samples were boiled in a metal bath at 99ºC for 10 minutes. Proteins were separated using SDS page gels and transferred to PVDF membranes (Bio-Rad, #1620177) for 3 hours using a wet transfer machine (Bio-Rad, #1703930). The membranes were blocked with 5% milk for 1 h at room temperature and then incubated overnight at 4°C with primary antibodies diluted with 5% BSA. CXCL1 (Invitrogen, #PA1-29220, 1:1000), PTX3 (Invitrogen, #PA5-101097, 1:1000), ITGA5 (Protein tech, #10569-1-AP, 1:1000), TIMP1 (Santa Cruz, #sc-21734, 1:1000), and β-actin (Santa Cruz, #sc-47778, 1:2000) primary antibodies were used in this study. After washing the membrane three times with TBST buffer, the membrane was incubated with secondary antibodies for 1 h at room temperature. Finally, blots were imaged using Bio-Rad Clarity ECL substrate (Biorad, #1705060) and ChemiDoc XRS+ System (Biorad).

### Enzyme-linked immunosorbent assay (ELISA)

Blood samples were collected in dry blood collection tubes, let stand for 1 h at room temperature. Samples were centrifuged at 3500 rpm for 15 min at 4ºC, then aliquoted and stored in -80ºC. CXCL1 (Elab Sciences, #E-EL-H0045), TIMP1 (Elab Science, #E-EL-H0184), ITGA5 (Boster Bio, #EK2227), and PTX3 (Invitrogen, #EH386RB) ELISA Kit were used to measure the concentration of each protein marker in serum. ELISA experiments were performed according to the user manuals from the manufacturer's protocol. Briefly, the standard curve was made by diluting the standard sample at different dilutions. Blank, samples and dilution of standard were added into the 96 well. Biotinylated antibody working solution were added and incubated for 1 hour at 37ºC. Decant the antibody and wash with 350 µl wash buffer for 3 times and pat it dry against clean absorbent paper. Then, HRP conjugated working solution were added to the well and incubated for 30 min at 37ºC. Wells were washed for 5 times using wash buffer. Add 90 µl Substrate Reagent to each well and incubated for 15 min, then 50 µl STOP solution were added. The absorbance of OD 450 nm was measured immediately using Tecan Spark plate reader.

### Statistical analysis

The R software package was used for bioinformatics analyses, as described above. For validation experiments, data were expressed as mean ± standard deviation (SD). Comparisons between groups were made using the student's t-test with statistical significance set at *P* < 0.05. Graphs were generated using GraphPad Prism 9.

## Results

### Identification of immune-related disease-causing genes

**Figure 1:**
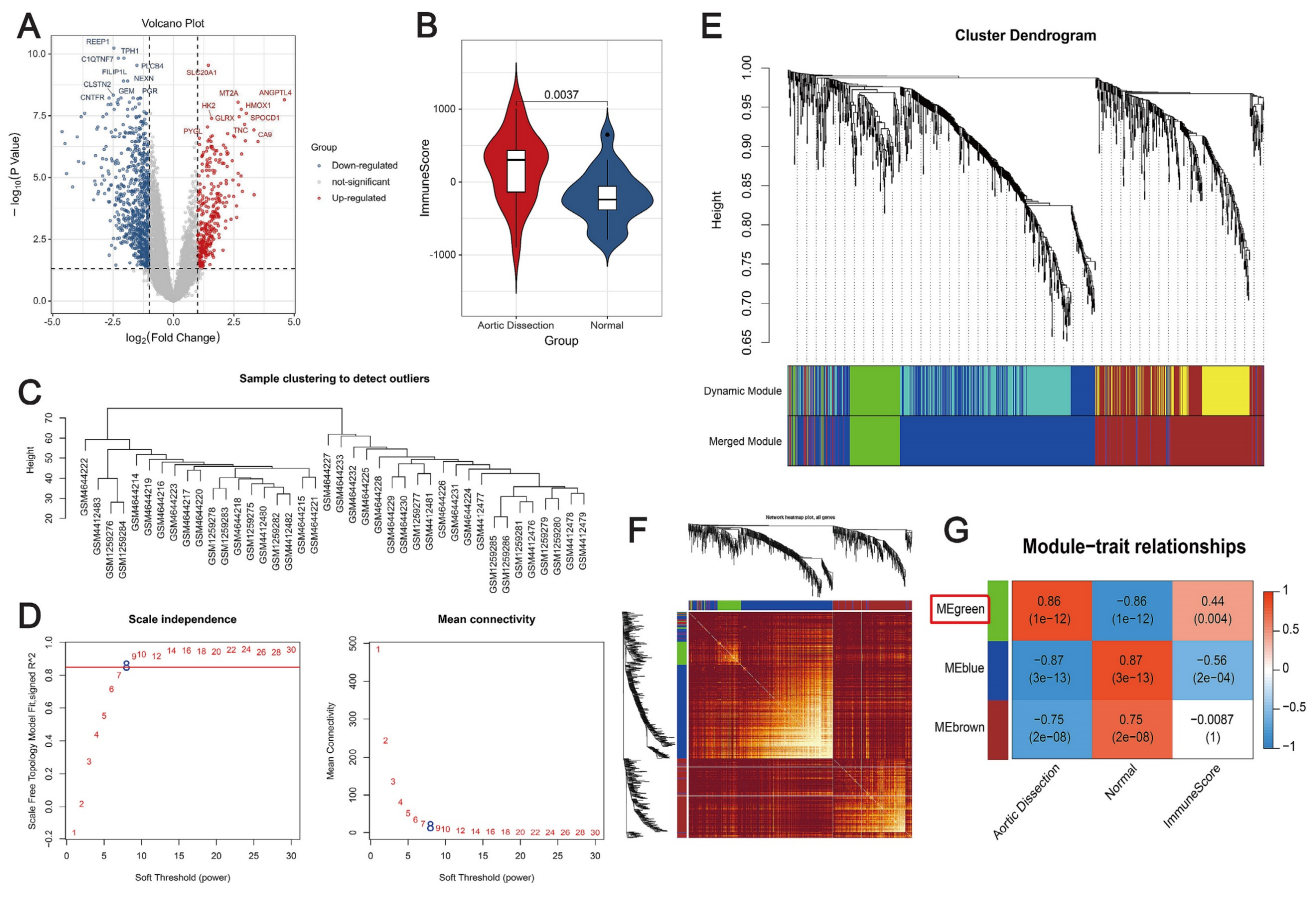
Co-expression analysis to identify immune-related pathogenic genes. (**A**) Volcano plot depicting changes in gene expression in unaffected and AD samples. Red and blue dots represent genes with up- and downregulated expression, respectively. (**B**) Violin plot showing differences in immune scores. Red, AD patients; blue, controls. (**C**) Dendrogram depicting sample distribution. (**D**) Plot of neighbor-joining matrix weights and neighborhood matrix weights. Left: plot of power parameters of selected neighborhood matrix weights. The horizontal axis represents the weight parameter, and the vertical axis represents the square of the correlation coefficient between log(k) and log(p(k)) in the corresponding network. Higher values of the squared correlation coefficient indicate that the network is closer to a scale-free distribution. The red line represents the critical value where the squared correlation coefficient reaches 0.85. Schematic representation of the average connectivity of genes under different power values of the neighborhood matrix weight parameters in the right panel. The red line represents the average connectivity under the power parameter selected in the left panel. (**E**) Tree diagram illustrating the division of gene co-expression modules. Different colors on the gene tree represent each module. (**F**) Heatmap showing the topological overlap matrix (TOM). Light yellow and dark red represent lower and higher TOM values, respectively. (**G**) Heatmap showing the correlation between individual modules and clinical features. The green module is strongly correlated with AD. Numbers in the upper and lower brackets represent correlation coefficients and *P*-values, respectively.

First, the DEGs between AD and control samples in the merged datasets were screened, and a total of 1131 DEGs were identified using the thresholds of false discovery rate (FDR) < 0.05 and |log_2_FC| ≥ 1 (Figure 1A). The immunity scores were significantly increased in AD patients compared with controls using the ESTIMATE algorithm (*P* < 0.05) (Figure 1B). Subsequently, WGCNA was performed to elucidate the relationship between the DEGs and immunity. We used the expression data of these genes, along with the immunity scores, as clinical features. The distribution of samples is shown in Figure 1C. To satisfy the requirement for a scale-free network distribution, we explored the values of the parameter “power” in the adjacency matrix. We defined a range of choices for the network construction parameters and computed the scale-free distribution topology matrix. As shown in Figure 1D, when the squared correlation coefficient reached 0.85 for the first time (indicated by the red line), we determined the value of “power”, i.e., “power = 8.” Using dynamic pruning (Figure 1E) and topological overlap matrix (TOM) clustering (Figure 1F), the 1131 genes were classified into three distinct modules. The correlation between each module and the clinical features was calculated, as shown in Figure 1G. After careful analysis, the green module was chosen for further examination. It exhibited a strong correlation with immunity and pathogenicity, boasting a correlation coefficient of 0.86 and a *P*-value of 1×10^-12^. Therefore, 136 genes in the green module, identified as targets associated with immunity and pathogenicity, were included in further analyses.

### Enrichment analysis and PPI networks

**Figure 2:**
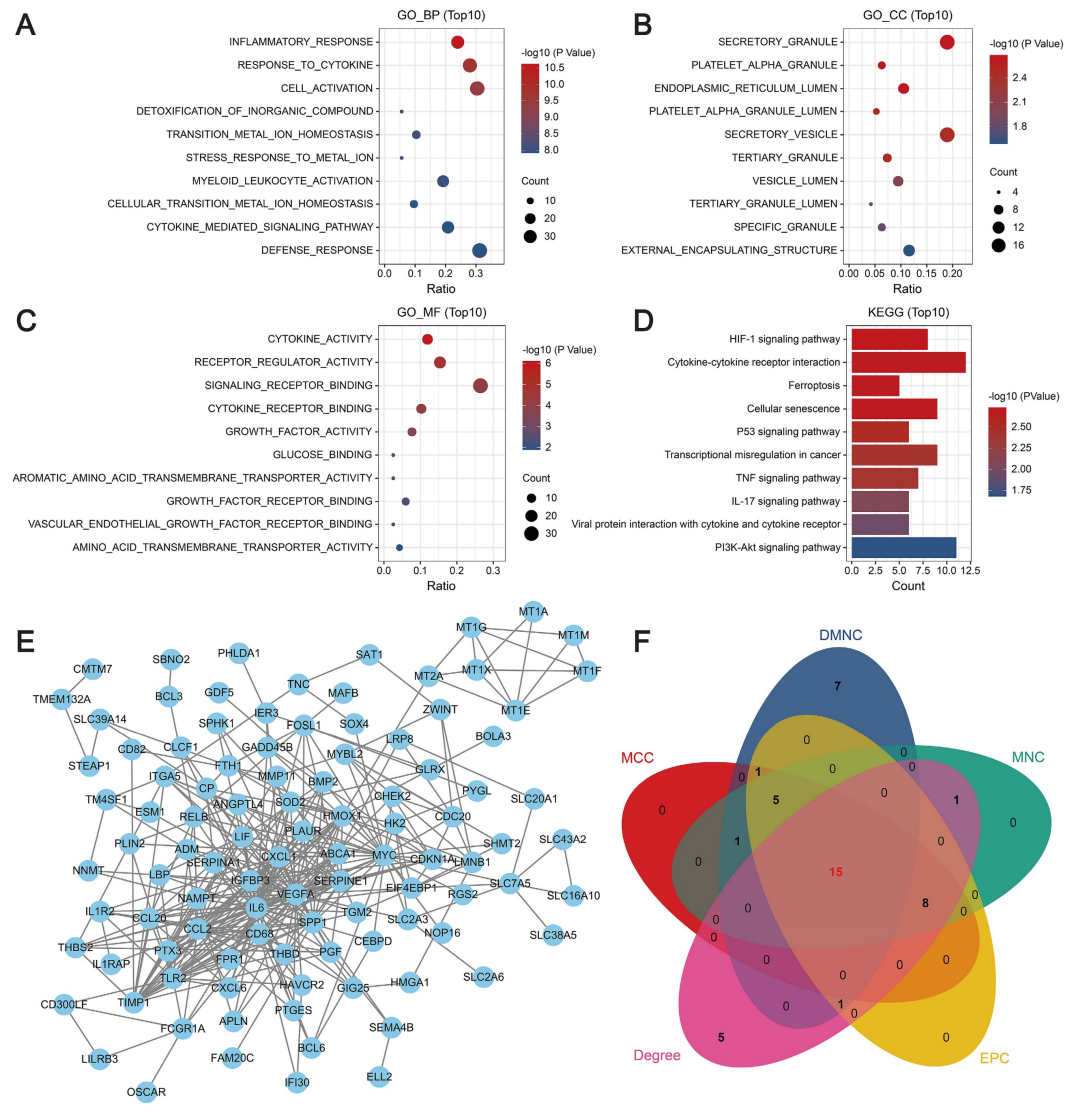
Molecular mechanisms of modular genes. (**A**-**C**) GO enrichment analysis: biological processes (A), cellular components (B), and molecular functions (C). The y-axis indicates different GO terms, the x-axis indicates the proportion of genes enriched in GO terms, the circle size corresponds to the number of genes, and the color represents the *P*-value. (**D**) KEGG pathway enrichment analysis: circle size represents the number of genes, and color represents the significance value. (**E**) PPI network revealing interactions between 136 genes. (**F**) Venn diagram showing the intersection of five topological analysis algorithms.

GO enrichment analysis revealed that the key genes were closely associated with biological processes such as the inflammatory response and the cytokine response, cellular components such as secretory granules and platelet α-granules, and molecular functions such as cytokine activity, receptor regulator activity, and signaling receptor binding. KEGG analysis revealed that the key genes were mainly related to the HIF-1 signaling pathway, cellular senescence, iron metabolism, cytokine-cytokine receptor interactions, and the p53, TNF, and IL-17 signaling pathways (Figure 2A-D). Then, a PPI network was constructed using the STRING database. In this network, proteins are represented by nodes, and the lines between the nodes indicate the presence of interactions between the proteins. This network revealed interactions between 136 of the genes, as shown in Figure 2E. We predicted and explored the top 30 important genes in the PPI network using five topological analysis algorithms, DMNC, MCC, MNC, Degree, and EPC, in the cytoHubba plug-in for Cytoscape. Through these analyses, 15 genes were identified as hub genes (Figure 2F).

### Machine-learning-based diagnostic modeling

**Figure 3:**
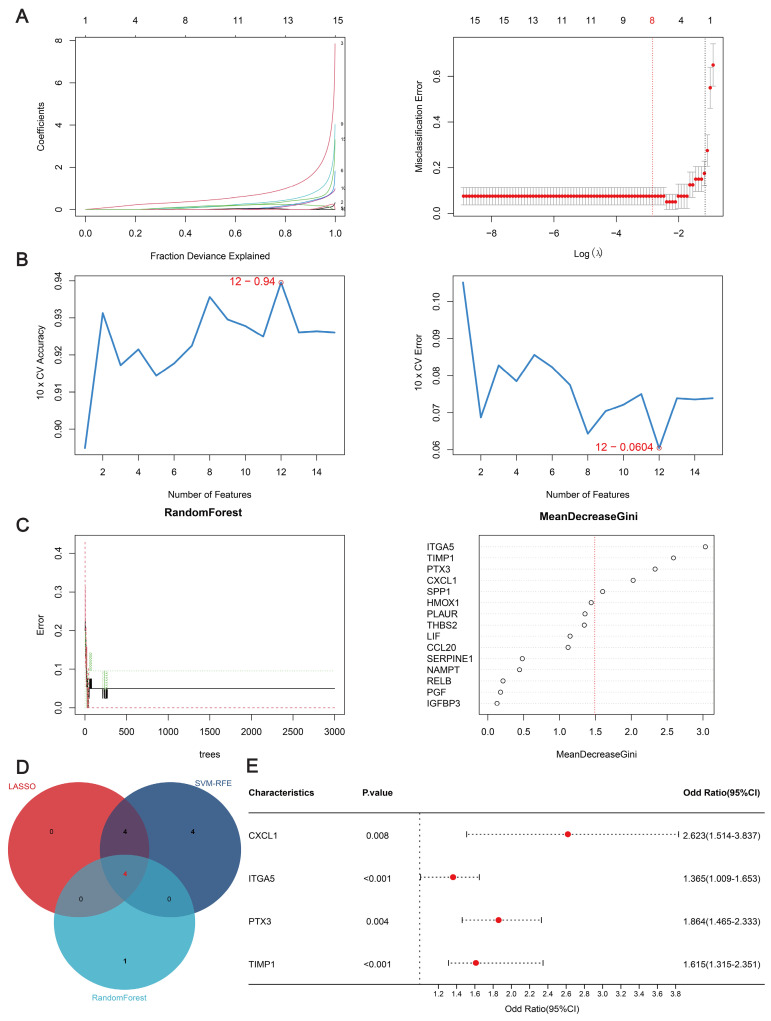
Machine learning algorithm for identifying feature genes. (**A**) Ten-fold cross-validation for selecting the best parameters for the LASSO model. Each curve represents a different gene. The figure shows the LASSO coefficient curve. The vertical solid line represents the partial likelihood deviation SE, and the dashed line is perpendicular to the optimal lambda. (**B**) SVM-RFE feature selection algorithm. The relationship between the number of trees and the error rate. Genes are ranked according to their relative importance. (**C**) Analysis of the number of trees, the error rate, and the relative importance ranking of genes by random forest. (**D**) Venn diagram of common feature genes identified by the LASSO, random forest, and SVM-RFE algorithms. (**E**) Forest plots of the four feature genes.

Three algorithms were used to identify feature genes from the 15 hub genes. For the LASSO algorithm, after ten rounds of cross-validation, we chose the smallest criterion with the highest accuracy to construct the LASSO classifier, which resulted in the identification of eight feature genes (Figure 3A). For the SVM-RFE algorithm, the minimum classifier error was achieved when the number of features reached 12 target genes (Figure 3B). According to the criterion of MeanDecreaseGini > 1.5, the Random Forest algorithm identified five significant feature genes (Figure 3C). The feature genes derived from the three algorithms were subjected to comparative analysis, leading to the identification of four pivotal feature genes—*CXCL1(*C-X-C motif chemokine ligand), *ITGA5* (Integrin Subunit Alpha 5), *PTX3* (pentraxin 3), and *TIMP1* (tissue inhibitor of metalloproteinases 1)—illustrated in Figure 3D. Then, multivariate logistic regression was used to calculate the regression coefficients of the four feature genes and their expression levels in the training dataset, which were then used to derive the diagnostic scores as follows:

Diagnostic Score = (*CXCL1* × 0.1525) + (*ITGA5* × 0.2043) + (*PTX3* × 0.1331) + (*TIMP1* × 0.0984)

As can be seen from the forest plot, *P* < 0.05 and odds ratio (OR) > 1 for all four of these characteristic genes, further confirming that these four genes are risk factors that may differ significantly between AD patients and unaffected individuals (Figure 3E).

### Validation and performance evaluation of the predictive models

**Figure 4:**
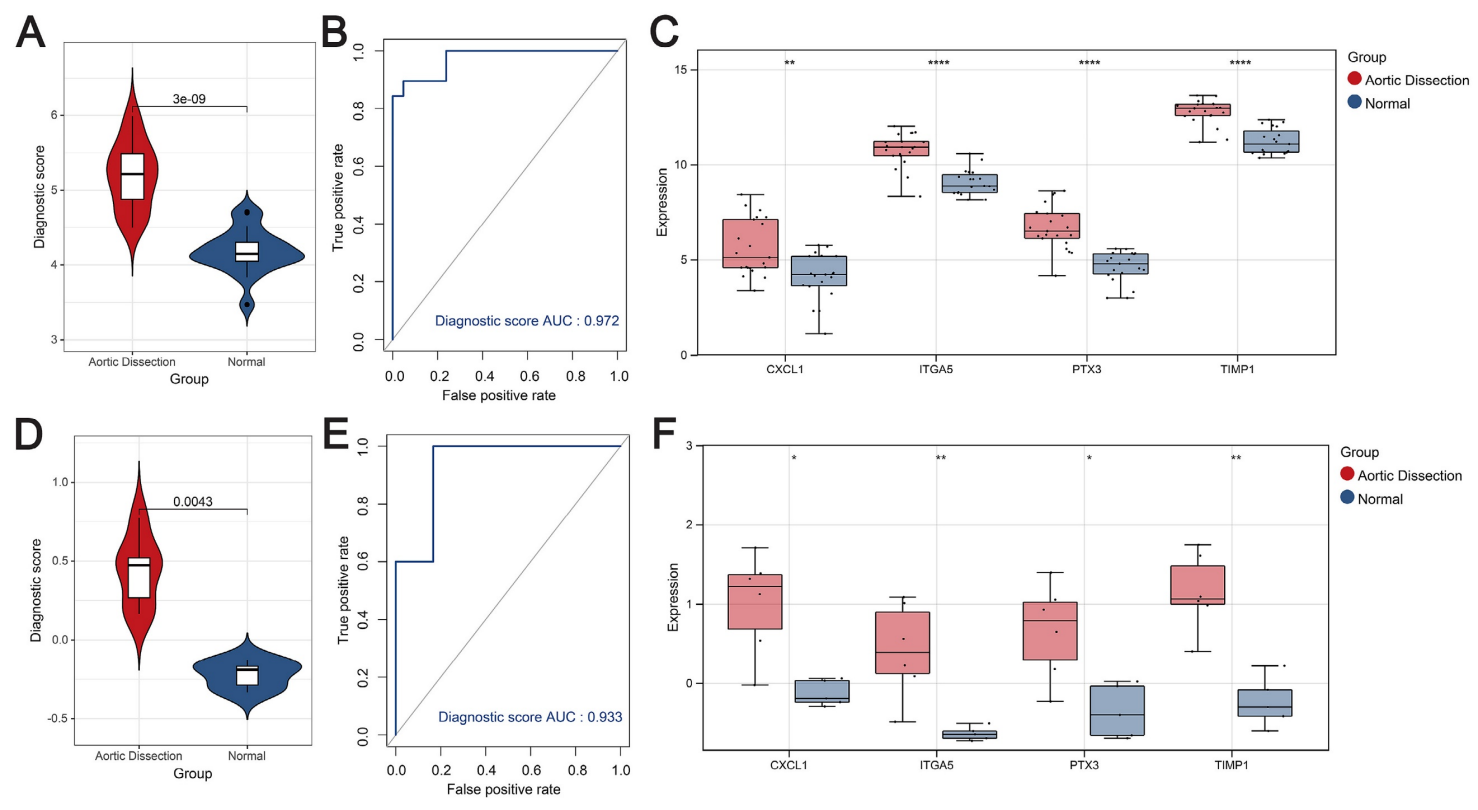
Diagnostic score performance assessment. (**A**-**C**) Violin plots showing the distribution of diagnostic scores (A), ROC curves illustrating the predictive performance of the diagnostic scores (B), and expression box plots showing the genetic profile of AD compared with the control population (C) in the training set. (**D**-**F**) Violin plots showing the distribution of diagnostic scores (D), ROC curves illustrating the predictive performance of diagnostic scores (E), and expression box plots showing the genetic profile of AD compared with the control population (F) in the external validation set.

The diagnostic scores constructed using the four hub genes were effective in differentiating between AD and the unaffected population. First, in terms of the training set, the scores for AD patients were significantly higher than those of the unaffected population (*P* < 0.05) (Figure 4A), and the AUC value of the ROC curve was higher, at 0.972 (Figure 4B). The diagnostic genes were significantly different between the AD and control populations (Figure 4C). Second, the diagnostic scores in the AD group were significantly higher than those in the unaffected population in the external validation set (GSE98770) (*P* < 0.05) (Figure 4D), and the ROC curve showed an AUC value of 0.933 for the model (Figure 4E). Expression box plots showed that the diagnostic genes were significantly different between the AD and control populations (Figure 4F). To further evaluate the prediction performance, another external dataset was employed to validate the model, results showed strong prediction ability of the four-gene model (Figure S2). Thus, these results show the excellent predictive performance of the diagnostic score.

### Constructing a nomogram

**Figure 5:**
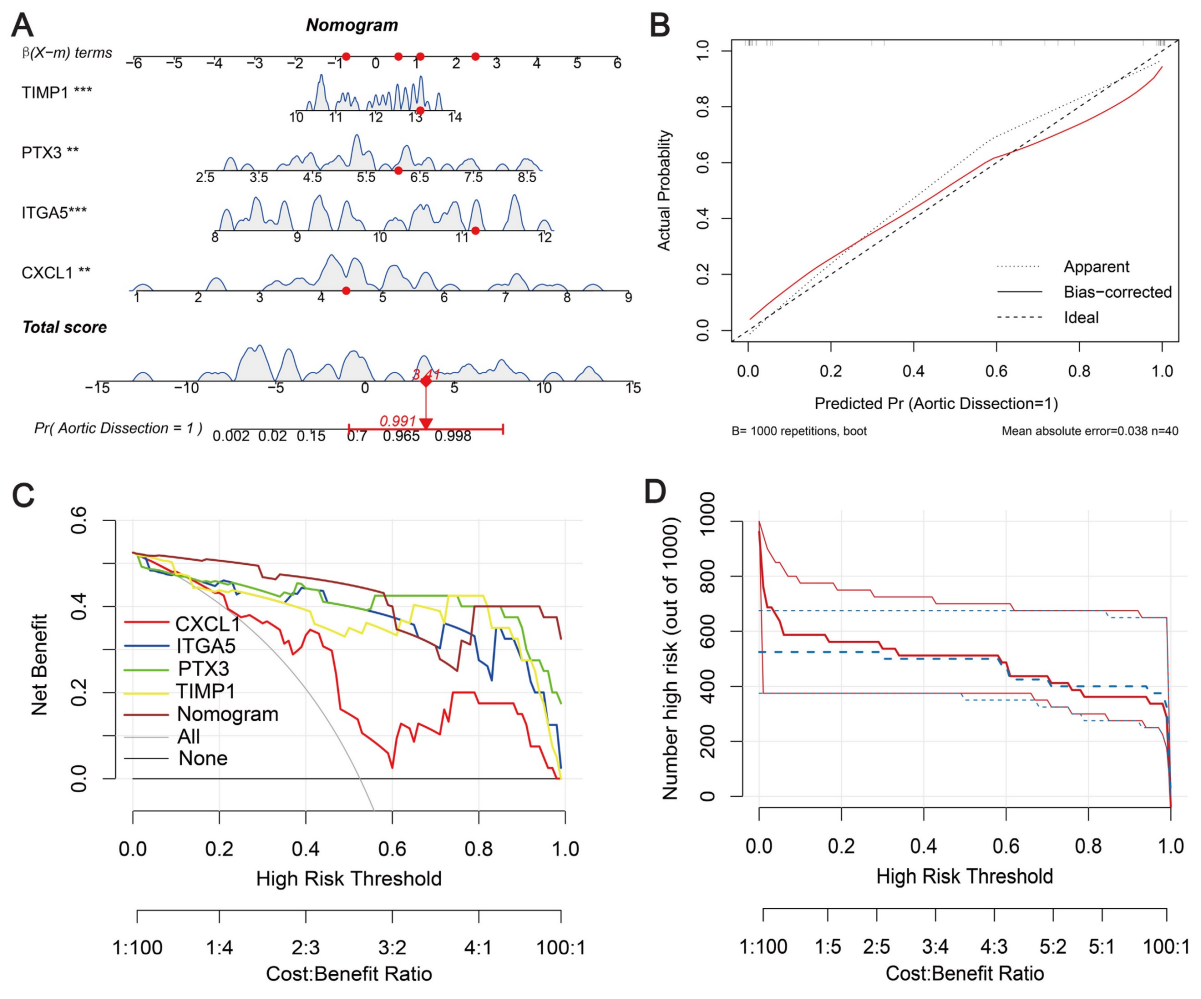
** Diagnostic nomogram**. (**A**) Predictive nomogram. (**B**) Calibration curve to assess predictive performance. (**C**) Decision curve analysis (DCA) for clinical assessment. (**D**) Construction of clinical impact curves from DCA results.

To validate the diagnostic ability of the four signature genes (*CXCL1*, *ITGA5*, *PTX3*, and *TIMP1*) for AD patients, we integrated them into a nomogram (Figure 5A). The calibration curve showed a small error between the actual and predicted survival risk, indicating high predictive accuracy (Figure 5B). Decision curve analysis (DCA) showed that the nomogram curve outperformed the grey line curve, indicating better clinical outcomes (Figure 5C). Clinical impact curves were constructed from the DCA results to visually assess the clinical impact of the nomograms. Notably, at a high-risk threshold of 0.3, the “number at risk” curve was very close to the “number at risk with events” curve, suggesting that the nomogram has good predictive power (Figure 5D). To evaluate the influence of demographic and clinical feature on the prediction accuracy, sex and age characteristics were added to the nomogram, as shown in Figure S3A. The calibration curve shows that the observed outcomes are consistent with the predicted probabilities (Figure S3B). This suggests that the nomogram exhibits good predictive power. The DCA curve shows better net benefit, suggesting that the nomogram has good clinical value (Figure S3C).

### Analysis of infiltrating immune cells

**Figure 6:**
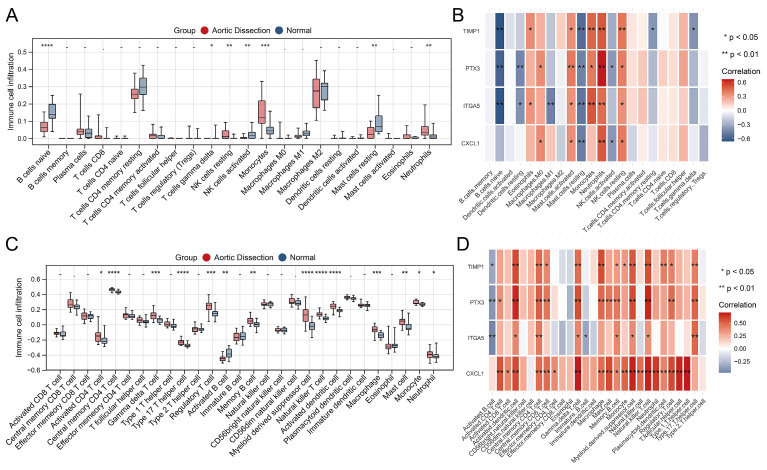
Analysis of immune cell infiltration in AD and unaffected controls. (**A**, **C**) Comparison plots illustrating changes in immune cell populations in AD and unaffected populations. (**B**, **D**) Heatmaps depicting the correlation between the four identified genes and infiltrating immune cells. *P < 0.05; **P < 0.01; ***P < 0.001. The horizontal axis represents the immune cell subtypes, and the vertical axis represents the key genes.

Using training set expression profiles, we employed the CIBERSORT and ssGSEA algorithms to determine the composition of the immune cell types in each sample. Subsequently, we compared the proportions of the various immune cells in AD and unaffected populations, as shown in Figure 6A and C. This analysis showed significant differences, particularly in monocytes, neutrophils, and natural killer (NK) cells. Significant associations between the four characteristic genes and various infiltrating immune cells were observed, and the most common co-related immune cells were neutrophils, resting NK cells, resting mast cells, activated mast cells, activated dendritic cells, central memory CD4 T cells, gamma delta (γδ) T cells, natural killer T cells, and myeloid-derived suppressor cells (Figure 6B and D). These results suggest that these commonly associated immune cells may serve as therapeutic targets for AD.

### The four hub genes were significantly elevated in AD patient samples

**Figure 7:**
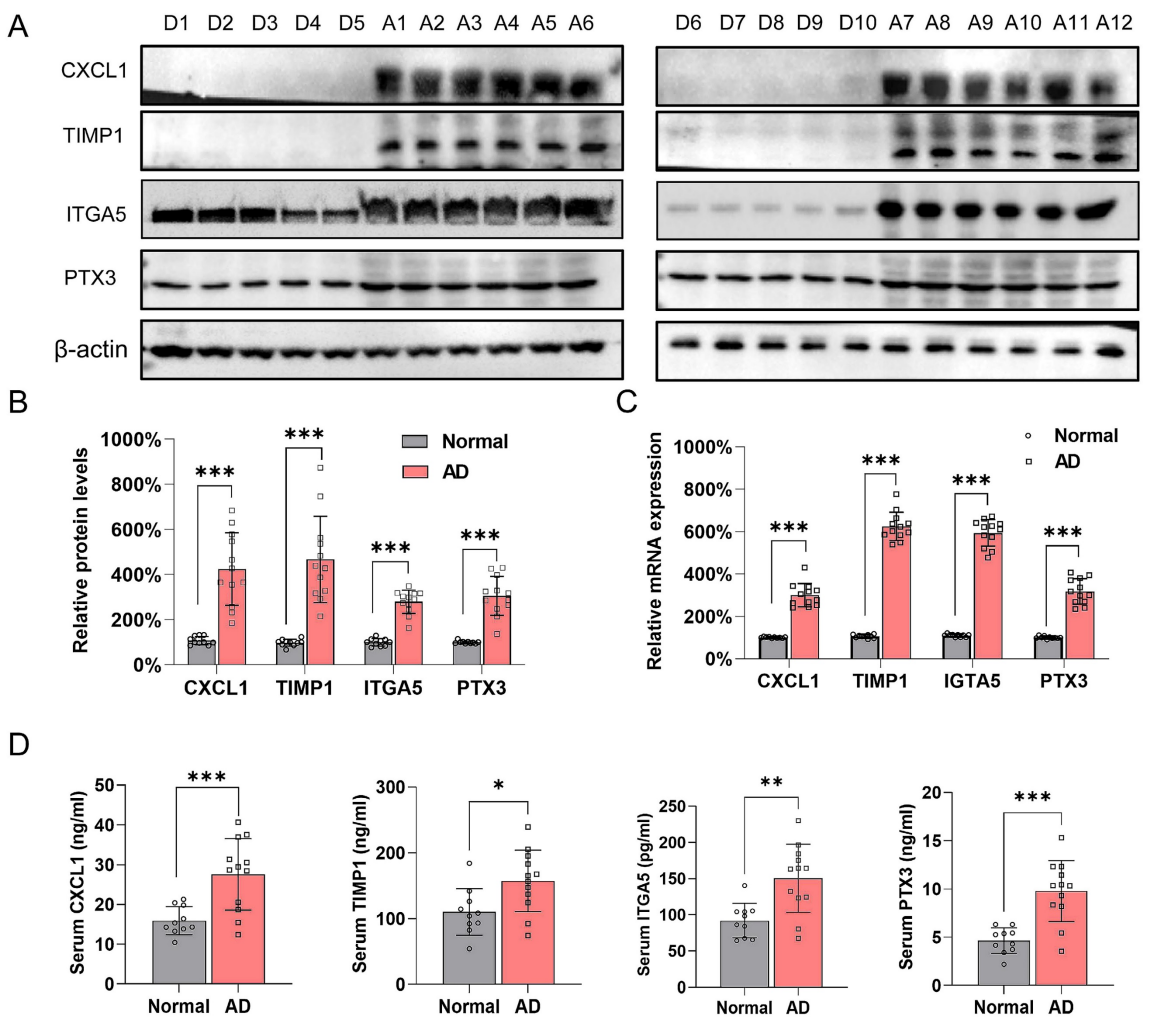
Validation of the four hub genes expression in control individuals and AD patients. (**A**-**B**). Protein level for CXCL1, TIMP1, ITGA5 and PTX3 in healthy donor (D1-D10) and AD patients (D1-D10) aortic samples. β-actin and Vinculin were used as internal control. ***P<0.001. (**C**) mRNA expression level of CXCL1, ITGA5, PTX3 and TIMP1 in healthy donors (D1-D10) and AD patients (A1-A12). β-actin was used as internal control. (**D**) Serum concentration of CXCL1, ITGA5, PTX3 and TIMP1 in healthy donors (D1-D10) and AD patients (A1-A12). *P < 0.05, **P < 0.01, ***P<0.001.

Samples from ten healthy donors were obtained from cadaveric donations (Fig. S4A). A total of 12 AD patients with complete medical records were involved in this investigation, and Fig. S4B shows representative CT angiography images of each patient (left) and the corresponding sample images (right). The protein levels of CXCL1, TIMP1, ITGA5, and PTX3 were much higher in the AD samples than in the control group (Figure 7A and B). The qRT-PCR results also supported this trend (Figure 7C). Furthermore, the expression pattern in patient samples were examined by ELISA. Results showed that the four hub genes were overexpressed in patient serum compared with control group (Figure 7D).

## Discussion

AD is a life-threatening cardiovascular disease that is difficult to diagnose because of its multifaceted clinical presentation. Although current imaging methods can accurately diagnose AD, they do not include the necessary biological information. The ability of machine learning to predict the risk of AD prevalence provides important support for medical research and clinical diagnosis by taking multiple factors into account, processing large-scale data, providing high-precision predictive capabilities, automated feature selection, and continuous optimization of models. Previously, several studies have been conducted to find biomarkers for the identification or diagnosis of AD and to explore its underlying mechanisms. Xu *et al.* found that some plasma metabolites, such as 1,5-anhydro-D-glucitol, D-(+)-sucrose, and PC (O-16:0/0:0), are associated with the risk of developing TBAD 28. Wan *et al.* found that MYC and ESR1 have diagnostic value and potential as biomarkers for age-related TAAD 29. Sadeghipour *et al.* identified a novel *EFEMP2* gene variant (c.C247T) associated with dominant non-syndromic thoracic aortic aneurysms 30. Wang *et al.* found that S100A8/A9, PTX3, and CHI3L1 could be practical tools for biomarker identification of an elevated risk of acute kidney injury after TAAD surgery 31. Huang *et al.* found that a combined CRP, d-dimer, and MMP9 detection model had the highest predictive value for one-year survival in acute type A aortic coarctation by ROC analysis 32. All these studies provide clues and directions for the prediction and diagnosis of AD.

In this investigation, we investigated gene expression levels in AD patients and unaffected controls using the GEO database, and a total of 136 genes were identified as target genes associated with immunity and pathogenicity for further analysis. Through GO and KEGG analysis, we identified key genes that are mainly associated with biological processes such as the inflammatory and cytokine responses, cellular components such as secretory granules and platelet α-granules, and molecular functions such as cytokine activity, receptor modulator activity, and signaling receptor binding. These genes are also closely related to pathways such as the HIF-1 signaling pathway, cellular senescence, the iron metabolic response, TNF and IL-17 pathways. It has been reported that HIF-1α upregulated CXCL1 through inducing miR-19a expression in endothelial cells (ECs)^33^. ITGA5 induction requires HIF-1 and HIF-2 under hypoxic 34, indicating close relationship between these genes and the identified signaling pathways. The enrichment of these biological functions and signaling pathways provides potential insights into the pathogenesis of AD. Finally, through LASSO, random forest, and SVM-RFE algorithms, four feature genes—*CXCL1*, *TIMP1*, *ITGA5*, and *PTX3*—were identified, and these four genes all showed upregulated expression levels in AD samples compared with controls. Consequently, a nomogram model using these four hub genes was constructed, which showed excellent signatures for predictive ability and diagnostic prospects. Two independent datasets and patient samples were utilized to validate the prediction model and performance.

The inflammatory response is included in AD progression 35. Hyperinflammatory patients with AD have worse outcomes than their hypoinflammatory counterparts 36. Vascular inflammation could by trigged by perivascular adipose tissue 37. Of note, the role of macrophages in AD has been deeply investigated 38, 39. It is well known that CXCL-1 and Matrix metalloproteinases are involved in IL-17 signature genes 40. Our current study reveals an increase of neutrophils, monocytes, resting NK cells, macrophages, type 2 and 17 T-helper cells, and γδ T cells in AD tissues, alongside a decrease in naive B cells, resting mast cells, and activated B cells. The four hub genes show partially similar but differentiated correlations with immune cells. The immune cells related to AD have been mentioned in previous reports 41-43. Our current research provides a spectrum of hypoxia and immune cell correlations related to AD, and provides new evidence supporting immune intervention by these four hub genes.

The major pathophysiological principle of aortic dissection is the increased pressure leading to the separation of layers of the media and creates a false lumen in the aortic wall. During this process, macrophage migrate and infiltrate into the aorta, neutrophils infiltration into the adventitia. While neutrophils are major cells that secrete MMP proteins, macrophages release ECM and mast cells also secrete MMPs, which jointly promote AD development 42, connecting AD to multiple immune markers, including the four hub genes in this study.

Firstly, CXCL1 levels is elevated in the aortic tissue and plasma, leading to an accumulation of neutrophils in the aortic wall. This infiltration contributes to tissue damage and destabilization of the aortic structure. Elevated CXCL1 were observed not only in aortic aneurysms in mice 44 but also in AD patients 45. The tunica adventitia of dissected aortas displayed high CXCL1 expression, facilitating neutrophil egress from the bone marrow and infiltration into the aortic adventitia, and in turn leading to local inflammation and aortic expansion and rupture 46. Secondly, TIMP1 is an important member of the metalloproteinase inhibitor family It plays a crucial role in regulating the activity of matrix metalloproteinases (MMPs) 47, 48. Inflammatory cytokines (such as TNF-α and interleukins) can increase MMP activity, leading to ECM degradation and resulting in aortic dissection. While TIMP1 regulates this process and participate the aortic wall instability. TIMP1 levels are found both elevated and decreased in patients with AD 49, 50. Besides, fibroblast-specific TIMP1 targeted intervention has shown therapeutic effects on experimental mouse AD 51. To keep a balance of TIMP1 and MMPs ratio might be valuable for AD prevention. Thirdly, ITGA5 refers to integrin subunit α5. it also plays a crucial role in the remodeling and maintenance of the ECM. Notably, the relationship between ITGA5 and AD has received limited attention in previous studies. Two studies found high expression of ITGA5 in AD tissues 52, 53. However, a reverse trend has also been found 54. Our study revealed that ITGA5 is significantly upregulated in AD tissues. IGTA might participated in AD progression via ECM remodeling. Lastly, PTX3 is a marker of vascular inflammation, it is rapidly upregulated during inflammation^55^. In aortic dissection, where there is significant vascular inflammation, may cause significant increase of PTX3. A previous study demonstrated that PTX3 was significantly overexpressed in ruptured tissues compared with stable abdominal aortic aneurysms 56. In addition, Seim *et al.* reported elevated levels of PTX3 in patients with Loeys-Dietz syndrome compared with other inherited thoracic aortic diseases 57. Furthermore, PTX3 has been associated with occasional pleural effusions in patients with acute AD 58. Taken together, the four hub genes might participate aortic dissection via neutrophils attraction, ECM remodeling and acute inflammation.

Although the four hub genes showed excellent diagnostic potentials in AD. They were reported to predict other disease. TIMP1 and CXCL1 has been reported to be elevated in colorectal cancer 59. CXCL1 and PTX elevation has been reported in acute myocardial infarction^60^. PTX3 and TIMP1 was involved in a glioma patients' diagnostic model^61^. However, the above study generated the prediction model combine with other hub genes. The combination of these four genes (*CXCL1*, *TIMP1*, *ITGA5*, and *PTX3)* has not been reported for disease prediction before, indicating its specificity for aortic dissection diagnosis.

This study has some limitations. Firstly, our data sourced from the public GEO database may introduce selection bias, despite being a common practice in bioinformatics. These datasets may not fully represent all AD cases due to biases in sampling and submission by original researchers. To mitigate this, we randomly sampled and maximized the number of datasets, ultimately selecting three for diversity and two external ones for validation. This approach reduced bias and improved model applicability, but potential bias could still affect generalizability. Secondly, while nomogram-based models are valuable for personalized prediction and decision-making, they may face challenges in resource-limited communities, where alternative methods like decision tree modeling could be useful. Additionally, we have identified signature genes but not fully elucidated their regulatory mechanisms in AD, necessitating further studies. Lastly, despite the model's good performance in validation, sample size limits its clinical effectiveness. Given AD's low incidence, multicenter collaboration is essential for collecting sufficient samples for future analysis.

## Conclusion

In this study, we utilized multiple machine learning algorithms to screen four immune-related genes—*CXCL1*, *ITGA5*, *PTX3*, and *TIMP1*—that exhibited distinct expression patterns between control and AD samples. Nomogram models constructed using these four genes demonstrated high predictive value for AD diagnosis. Further infiltrating immune cell analysis revealed that these genes are implicated in TNF, HIF-1, and IF-17 signaling pathways and are associated with some types of immune cells. Notably, these four hub genes were overexpressed in both patient tissues and serum samples. Our findings present a viable model for AD diagnosis and immunomodulation assessment.

## Supplementary Material

Supplementary figures.
