# Supplementary material for: Establishment of a nomogram model based on immune-related genes using machine learning for aortic dissection diagnosis and immunomodulation assessment
Source: Int J Med Sci. 2025 Jan 21;22(4):873–86. doi: 10.7150/ijms.100572 (PMC11843136; doi:10.7150/ijms.100572)
Supplement: Supplementary file 1 — Supplementary figures. [file ijmsv22p0873s1.pdf]

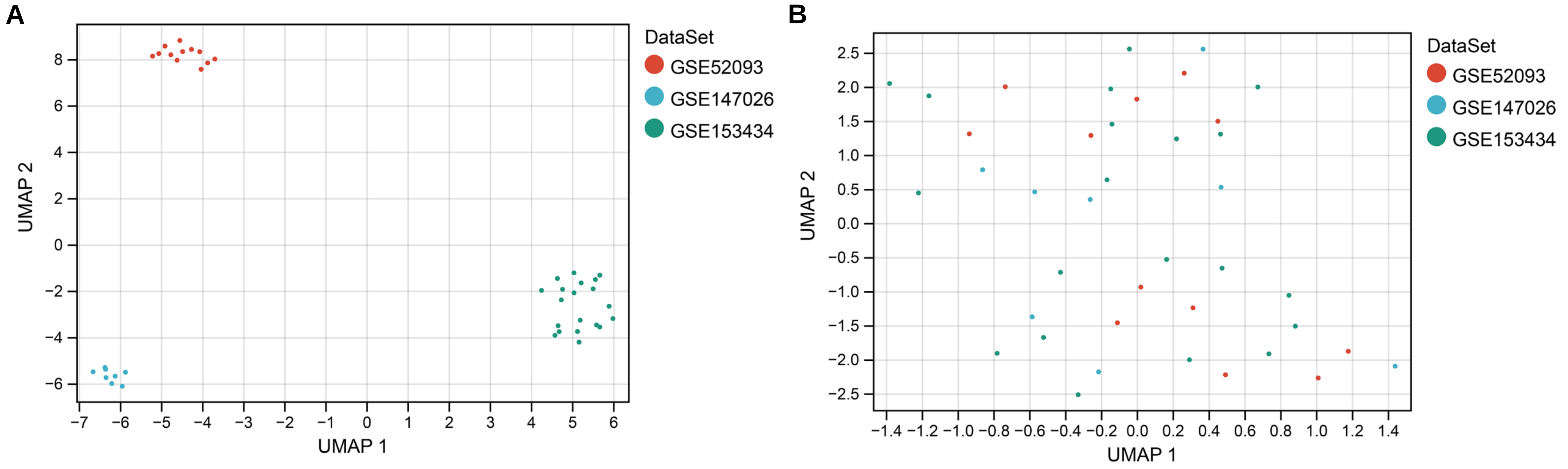

Figure S1. Diagrams of the samples before (A) and after (B) de-batching.

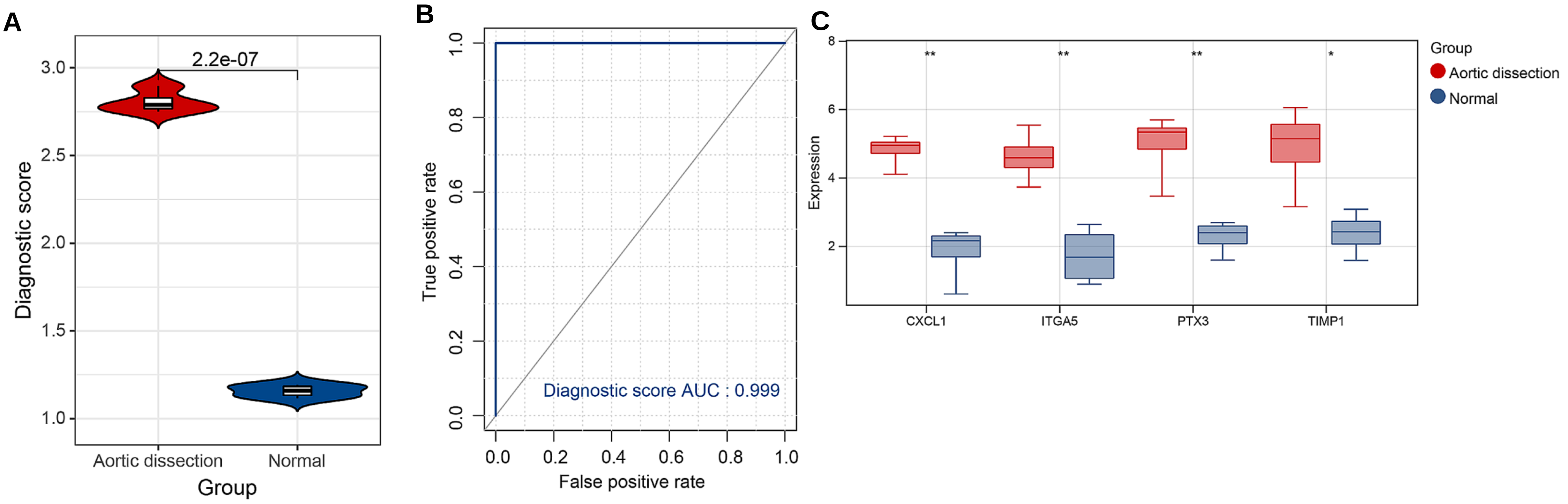

Figure S2: Performance evaluation of the diagnostic model using external validation dataset GSE190635.
(A) Diagnostic score distribution violin diagram of aortic dissection and healthy population. (B) Predicted ROC curve of diagnostic score. (C) Expression box diagram of the four hub genes.

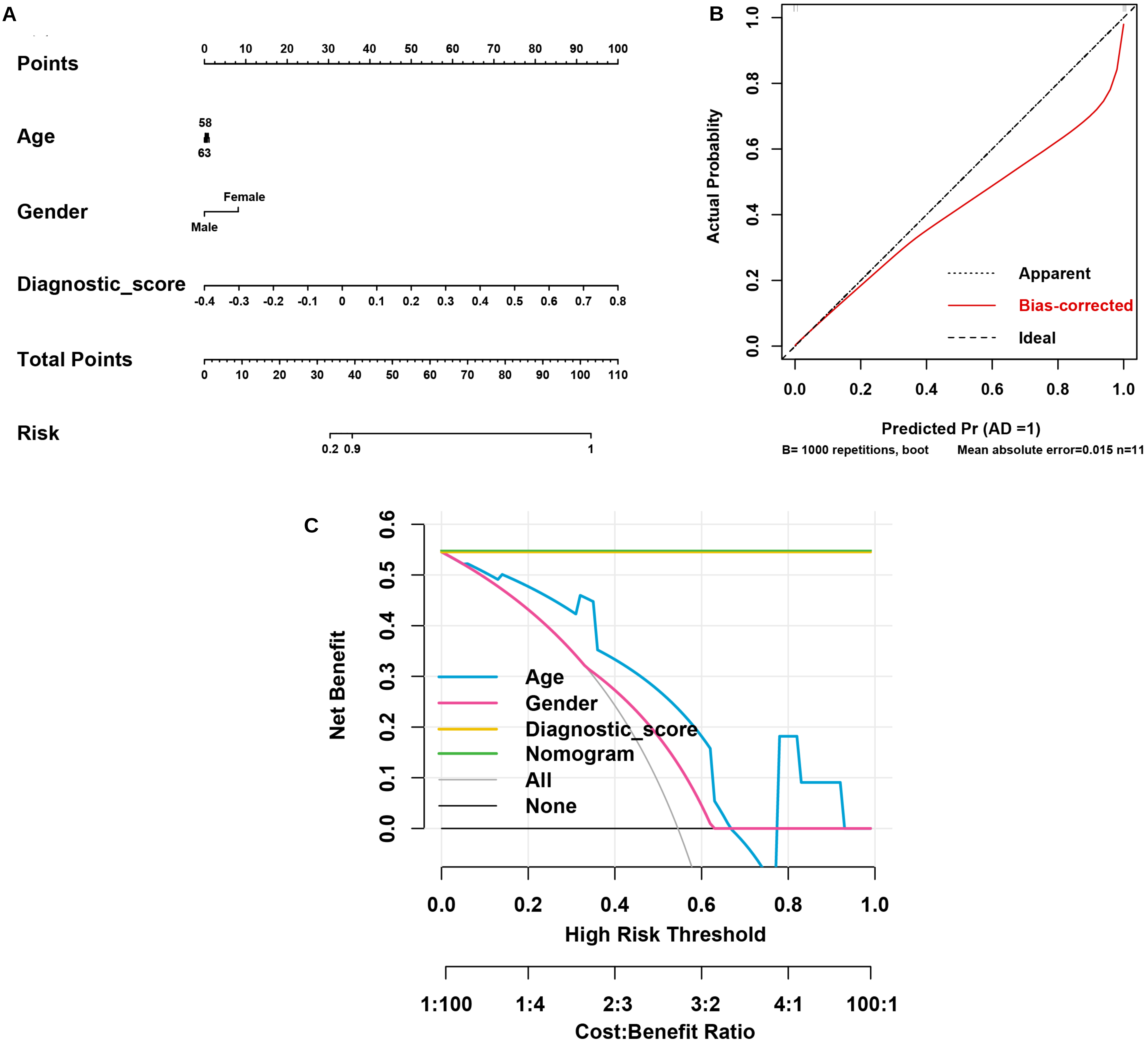

Figure S3. Clinical parameter nomogram.

(A). Nomograms of age, gender, and diagnostic scores. (B). Calibration curve used to evaluate the nomogram. (C). The Deca curve assesses the clinical value.

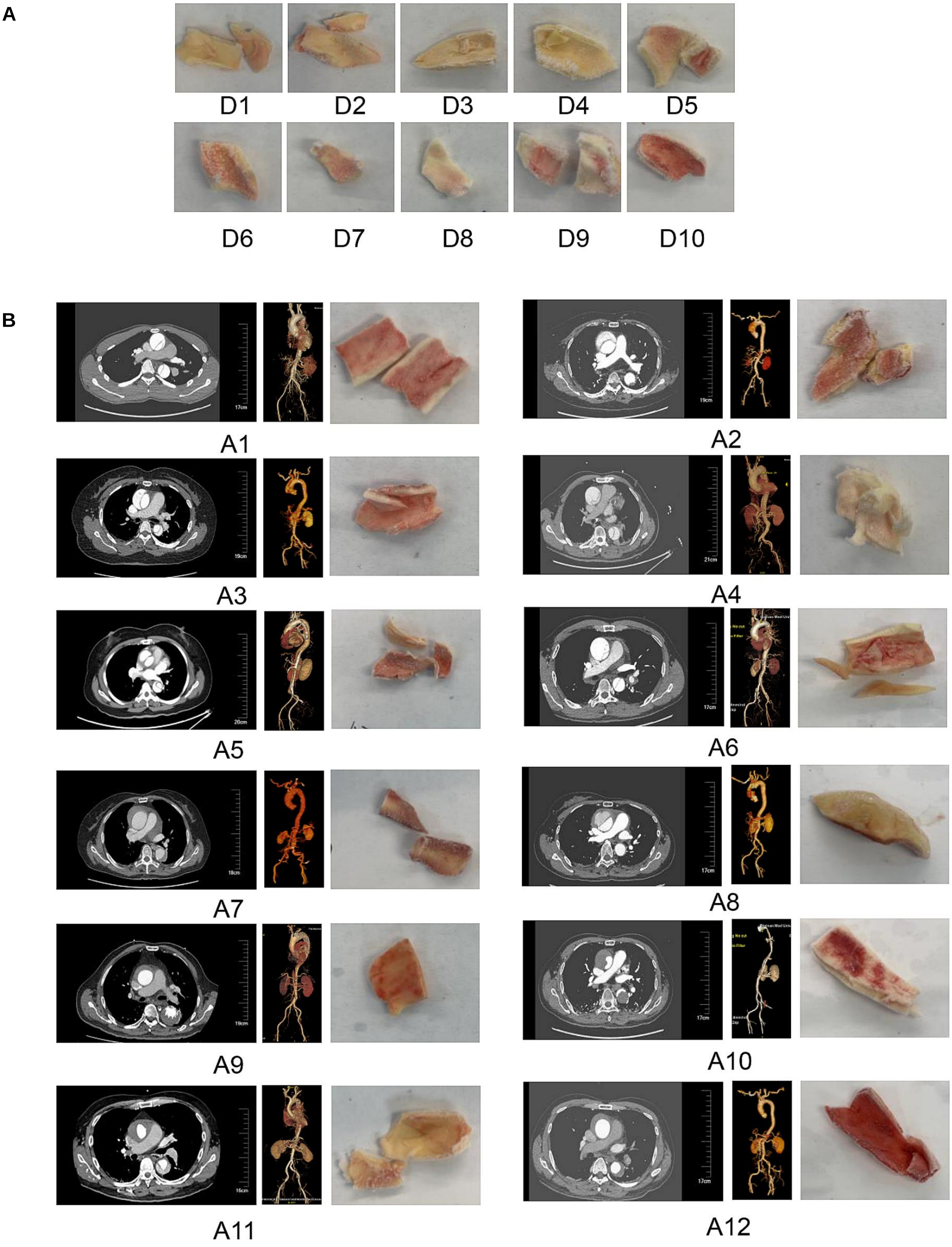

Figure S4. Sample images and angiography images for patients.
(A) Sample images from unaffected donors. (B) Paired CT angiography images and sample images of patients who were diagnosed with AD (A1–A12).
